# Pharmacological Treatment Strategies for Postpartum Depression

**DOI:** 10.1192/j.eurpsy.2022.1438

**Published:** 2022-09-01

**Authors:** O. Vasiliu

**Affiliations:** Dr. Carol Davila University Emergency Central Military Hospital, Psychiatry, Bucharest, Romania

**Keywords:** Antidepressants, postpartum depression, Brexanolone

## Abstract

**Introduction:**

Postpartum depression (PPD) is an important cause of discomfort and dysfunction that impair the quality of life and the daily functionality not only of the patient but also of her child and her family, in its entirety. New treatment options have been made available for this pathology, but their use is restricted by methodological aspects, like the difficulty of administration, lack of enough data regarding their long-term efficacy, and costs.

**Objectives:**

To conduct a literature review in order to find the most evidence-based pharmacological interventions for PPD.

**Methods:**

A literature review was performed through the main electronic databases (PubMed, CINAHL, SCOPUS, EMBASE) using the search paradigm “postpartum depression” AND “treatment” OR “pharmacological agents”. All papers published between January 2000 and August 2021 were included.

**Results:**

Among the most evidence-based agents for PPD treatment are serotonin selective reuptake inhibitors (SSRIs). As individual agents, sertraline seems to be the most supported antidepressant by evidence from clinical trials, followed by escitalopram/citalopram, and fluoxetine. Other antidepressants supported by clinical data were venlafaxine, desvenlafaxine, nortriptyline, and bupropion. A 6-12 months maintenance treatment is considered optimal after remission, in women with a low risk of recurrence. Brexanolone, zuranolone, and ganaxolone are members of a new class of drugs studied for postpartum depression, but currently, only the first agent is FDA-approved for this indication.

**Conclusions:**

SSRIs are the most supported by evidence treatments for PPD, and brexanolone is a drug with a new mechanism, dedicated to this pathology that provides new hope for recovery.

**Disclosure:**

No significant relationships.

